# Engineering Surfaces with Immune Modulating Properties of Mucin Hydrogels

**DOI:** 10.1021/acsami.1c19250

**Published:** 2022-08-24

**Authors:** Kun Jiang, Xueyu Wen, Torbjörn Pettersson, Thomas Crouzier

**Affiliations:** † Division of Glycoscience, Department of Chemistry, School of Engineering Sciences in Chemistry, Biotechnology and Health, KTH, Royal Institute of Technology, AlbaNova University Center, Stockholm 106 91, Sweden; ‡ AIMES - Center for the Advancement of Integrated Medical and Engineering Sciences at Karolinska Institutet and KTH Royal Institute of Technology, Stockholm SE-100 44, Sweden; § Department of Neuroscience, Karolinska Institutet, Stockholm SE-171 77, Sweden; ∥ Division of Fibre Technology, Department of Fibre and Polymer Technology, School of Engineering Sciences in Chemistry, Biotechnology and Health, KTH Royal Institute of Technology, Stockholm SE-100 44, Sweden

**Keywords:** biomaterials, mucin coating, immune-modulating, macrophage polarization

## Abstract

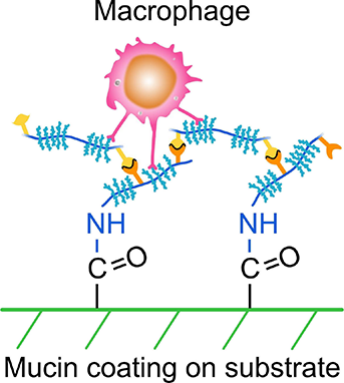

Hydrogels of cross-linked mucin glycoproteins (Muc-gel) have shown strong immune-modulating properties toward macrophages in vitro, which are translated in vivo by the dampening of the foreign body response to implantation in mice. Beyond mucin hydrogels, other biomaterials such as sensors, electrodes, and other long-term implants would also benefit from such immune-modulating properties. In this work, we aimed to transfer the bioactivity observed for three-dimensional Muc-gels to the surface of two model materials by immobilizing mucin into thin films (Muc-film) using covalent layer-by-layer assembly. We tested how the surface immobilization of mucins affects macrophage responses compared to Muc-gels. We showed that Muc-films on soft polyacrylamide gels mimic Muc-gel in their modulation of macrophage responses with activated gene expression of inflammatory cytokines on day 1 and then dampening them on day 3. Also, the markers of polarized macrophages, M1 and M2, were expressed at the same level for macrophages on Muc-film-coated soft polyacrylamide gels and Muc-gel. In contrast, Muc-film-coated hard polystyrene led to a different macrophage response compared to Muc-gel, having no activated expression of inflammatory cytokines and a different M1 marker expression. This suggested that the substrate mechanical properties and mucin molecular configuration determined by substrate–mucin interactions affect mucin immune-modulating properties. We conclude that mucin immune-modulating properties can be transferred to materials by mucin surface immobilization but will be dependent on the substrate chemical and mechanical properties.

## 1 Introduction

Biomaterials play an essential role in medicines by acting as building blocks for medical devices, tissue engineering scaffolds, and immunotherapies.^1−3^ However, the development of biomaterials has been limited by the adverse immune responses to implanted biomaterials.^4^ Macrophages are the central player in the immune system, and their responses mediate the innate and the adaptive immune systems.^5,6^ Macrophages have complex receptors sensitive to both chemical^7^ and physical^8^ stimulations, which are reflected by their phenotypic plasticity.^9^ Therefore, the chemical, mechanical, and biological properties of biomaterials, even in their finest details, could affect macrophage responses. This also suggests that by carefully tailoring the physical and chemical properties of biomaterials, one could direct their immune-modulating properties in the direction that is most favorable for the indicated treatment.^6^

We and others have shown that mucin-based hydrogels exhibit such immune-modulating properties.^10−12^ Covalently cross-linked bovine submaxillary mucin (BSM) hydrogels (Muc-gel) could dampen the complementary activation when exposed to human blood^13^ and dampen the recruitment of innate and adaptive immune cells toward the hydrogel in vitro^14^ and when implanted in vivo.^10^ The abundant glycan side chains of mucin glycoproteins can act as ligands to surface receptors on immune cells with downstream effects on cell inflammatory signals.^15,16^ For instance, binding of the Siglec-9 receptor of macrophages to sialic acid led to decreased expression of proinflammatory cytokines.^17^ However, hydrogels are not the only materials to be subjected to such immune responses. Hard metal, ceramic, and polymer-based implantable biomaterials, which offer complementary functions and performance toward hydrogels, would also benefit from the immune-modulating properties exhibited by mucin hydrogels.^18−20^ Assuming that Muc-gels owe their bioactivity mainly to the chemical signaling toward cells occurring at their surfaces, one could, in principle, cover any biomaterial with mucins to achieve similar bioactivities with potential application for translating immune-modulating functions to other implantable biomaterials.

In this work, we test whether the immune-modulating bioactivities of Muc-gels can be translated to near-2D coatings. We explore several determining parameters for mucin bioactivity at the surface. It has been shown that strategies used to bind mucin to surfaces can affect the coating stability,^21,22^ the molecular configuration of mucins at the surface,^23,24^ and then the accessibility of binding sites to bacterial adhesins.^25^ When exposed to friction,^21^ covalently grafted mucin coatings stayed hydrophilic for 180 days,^22^ which was more stable than passively adsorbed mucin coatings. The grafting strategies of coatings can also affect the configuration of the mucin molecules at the surface. For instance, more hydrophobic areas of mucins were exposed when covalently grafted to the surface via their amine groups and the coating was more sensitive to microenvironment changes than the physically adsorbed coatings.^23^ The coating stability and molecular configuration then affected the binding of mucin-specific antibodies and probiotics to the surface.^25^ We have also demonstrated that the molecular configuration of mucin in hydrogels can modulate macrophage responses.^26^ Along with the grafting strategies, the stiffness of the substrate onto which mucin is coated can also regulate macrophage responses.^8,27,28^

**Figure 1:**
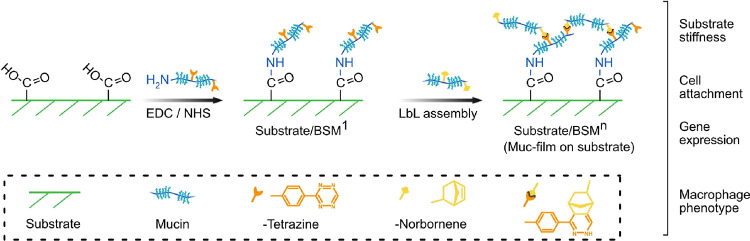
Illustration of the covalent mucin coating and LbL assembly for multilayers on different material surfaces. The monolayer mucin-coated surface is labeled as substrate/BSM^1^ (substrate is PS or PAAm in this study), and the “/” represents the covalent linkage between the substrate and mucin. After LbL assembly, the superscript “*n*” of BSM represents the amount of mucin layers assembled on the surface, and the “Muc-film” represents the multilayered mucin coating in this paper.

To test these critical parameters, we coated mucin on substrates via stable covalent linkages and assembled a pure mucin thin film via layer-by-layer (LbL) assembly (Figure 1). We hypothesize that such a multilayered mucin coating could help mimic the mucin molecular configuration at the surface of the material.^29−31^ To investigate the effects of substrate stiffness, polystyrene (PS, *E* = 354 ± 119 MPa) was used as a “hard” model substrate and polyacrylamide hydrogel (PAAm, *E* = 13 ± 2.5 kPa) was used as a “soft” model substrate with a similar stiffness to that of mucin hydrogels (*E* = 32 ± 1.8 kPa) (Table S2). We found that stable multilayered mucin coatings (Muc-film) can be assembled through covalent bonds on soft PAAm hydrogels and hard PS substrates. The Muc-film on PAAm conferred the materials similar macrophage responses as those of Muc-gel, while Muc-film on the hard PS substrate showed more limited effects.

## 2 Results and Discussion

### 2.1 Stability of the Covalently Grafted Mucin Coating and Muc-film on Polystyrene and Polyacrylamide Hydrogels

**Figure 2:**
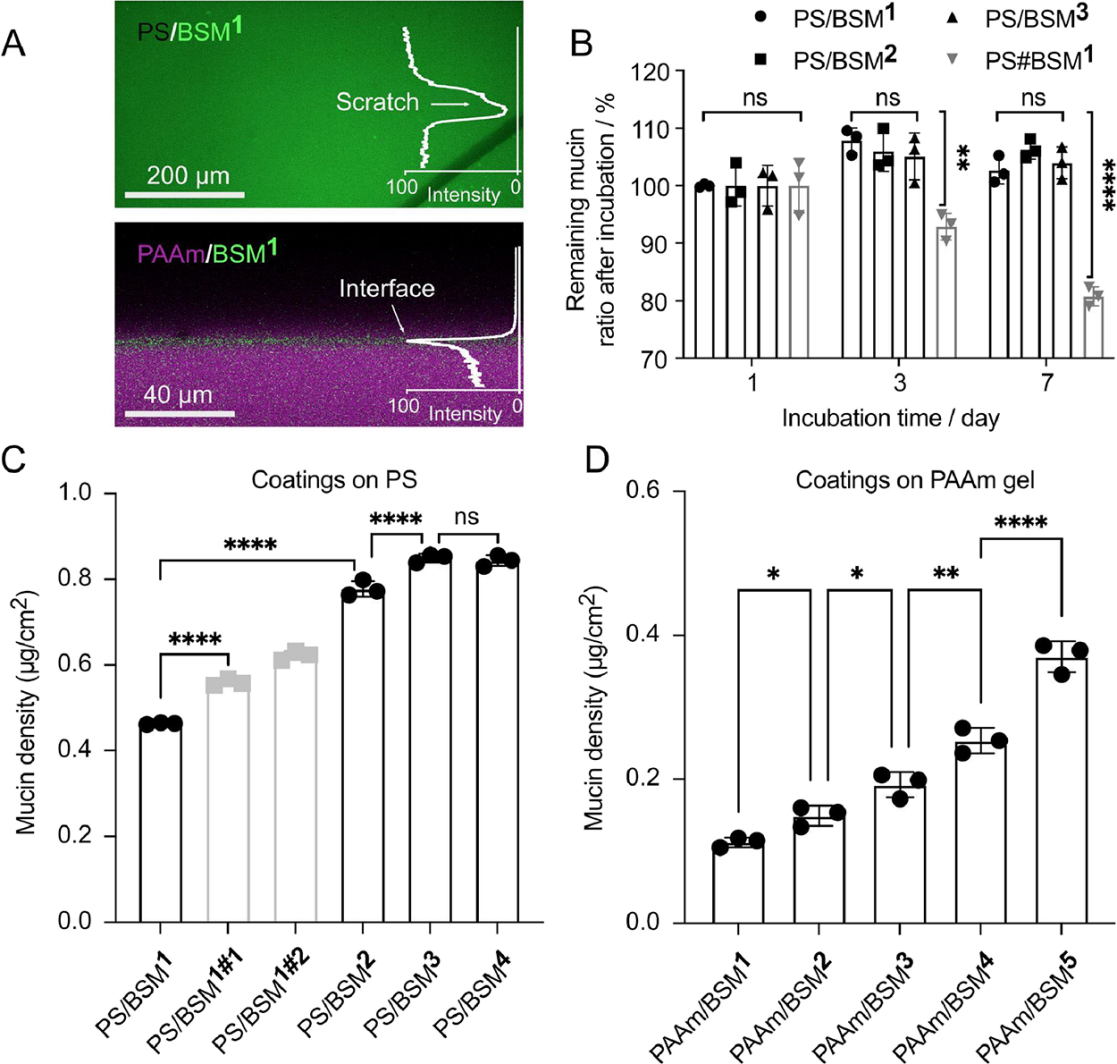
(A) Fluorescent images of mucin-coated PS (scratch shown on the bottom right to confirm mucin coating) and cross section of mucin-coated PAAm. The inserted curves on the right of the images are the normalized fluorescent intensity across the scratch on PS/BSM^1^ or the PAAmBSM^1^ interface. (B) Stability of mucin coatings after washing with PBS daily over 1 week. (C, D) Calculated mucin amount (or mass) of Muc-films after LbL assembly on PS (C) and PAAm (D). The “/” indicates the covalent bond and the “#” indicates the physical interactions of mucin–substrates or mucin–mucin. The superscript “*n*” of BSM represents the number of mucin layers assembled via covalent bonds on the surface, and “#” between the “*n*” indicates multilayer assembly without a click reaction. Statistical significance was calculated by a one-way ANOVA test by Prism (9.0). *, **, ***, and **** indicate *p* values of <0.05, 0.01, 0.0005, and 0.0001, respectively.

Mucin is a bottlebrush-structured glycoprotein (Figure 1) composed of a protein “core” and oligosaccharide “brush”. This complex structure enables mucins to be adsorbed passively on both hydrophilic^23,32^ and hydrophobic materials.^21,33^ However, it was reported that passively adsorbed mucin coatings can be desorbed by the friction occurring in the human body.^21,22^ To stabilize mucin at the material surfaces, we employed a more stable covalent grafting strategy. We used carbodiimide chemistry to graft BSM on PAAm or PS modified to exhibit carboxylic acid groups at their surfaces (Figure 2A). We confirmed that close to 100% of covalently grafted mucin coatings remain after daily PBS washing for one week, while only 80% of mucin from passively adsorbed coatings remain (PS#BSM^1^, Figure 2B). This result is in agreement with the work of Winkeljann et al., which reported that mucin covalently grafted to PMMA surfaces resisted desorption by ultrasonic treatment, while approximately 40% of passively adsorbed mucins were removed.^21^

Mucin bioactivity depends greatly on their interactions with cell surface receptors,^23,34^ such as Siglec receptors binding to sialic acid-containing glycans.^15,35^ By overexpressing and binding to sialic acid, the Siglec receptors are involved in both inflammatory signal activation and inhibition.^36,37^ The anchoring of mucin to the surface could change the molecular configuration of mucin and affect which moieties are exposed or confined to the surface, possibly leading to changed ligand accessibility to cell surface receptors. In contrast, the covalently cross-linked Muc-gels, which have shown strong immune modulation in vivo and in vitro, provide a random arrangement of mucins in the bulk and at the surface, where it is less likely to hide specific ligands.^10,14,26^ To mimic as closely as possible the molecular assembly of Muc-gel at the surface, Muc-films were built by LbL through the same click reaction between tetrazine and norbornene-functionalized BSM as previously reported (Figure 1). We determined by qNMR that each BSM molecule had more than 100 tetrazine or norbornene groups (Figure S1 and Table S1), which suggested that each molecule had the capacity to form several cross-linking bridges with several counterpart mucin molecules (Figure 1).

The amount of mucin in the multilayer assembly on PS and PAAm was higher than that for single-layer grafting (Figure 2C,D). It stopped increasing after three layers on PS, while no limit was reached on the PAAm surfaces onto which up to five layers could be assembled. The addition of mucins to a pre-adsorbed mucin coating led to a moderate increase in the amount of mucins at the surface even without the click reaction between the layers (Figure 2C, PS/BSM^1#1^ and PS/BSM^1#2^), which indicated that there was still some parts of the uncoated surface exposed to mucin after washing between the layer assembly. The density of mucin coatings on PS was significantly higher than that on PAAm with 0.85 μg/cm^2^ for three layers on PS (PS/BSM^3^) and 0.19 μg/cm^2^ for three layers on PAAm (PAAm/BSM^3^). Since the three-layer mucin coatings (Muc-film) share the same cross-linking chemistry with Muc-Gels, they might also exhibit similar mucin configuration at their surface, and were thus tested as possible two-dimensional mimics of Muc-gel in the rest of this study.

The high grafting densities on PS could be explained by the hydrophobic interaction between PS and the mucin protein core, which bring in close proximity amine groups of mucins and the carboxylic group at the PS surface and favor the carbodiimide coupling. The electrostatically neutral and hydrophilic surface of PAAm limits protein adsorption,^38^ while the negatively charged acrylic acid co-polymerized within PAAm could repulse the negatively charged mucins. The weak adsorption or repulsion from the surface of the gel could explain the limited efficacy of the carbodiimide coupling. The limits in the multilayer growth might be due to different accessibilities of the tetrazine or norbornene groups. It is possible that the strong hydrophobic character of the PS induces a re-conformation of the mucin, allowing the more hydrophobic character of the mucin backbone to get closer to the PS surface and allowing the tetrazine or norbornene groups to covalently graft the mucin to the surface. Meanwhile, electrostatic repulsion between the negative charges (due to the presence of co-polymer AA) of PAAm and mucin would lead to gels containing more flexible mucin conformation. The observed differences between the two surfaces in how mucin adsorbes to the surface and then assembles into an LbL films suggest different molecular configurations of the mucins on, which could affect their interaction with ligands such as cell surface receptors.

### 2.2 Muc-film Confers the Surfaces Cell Repulsive Properties of Muc-gels

**Figure 3:**
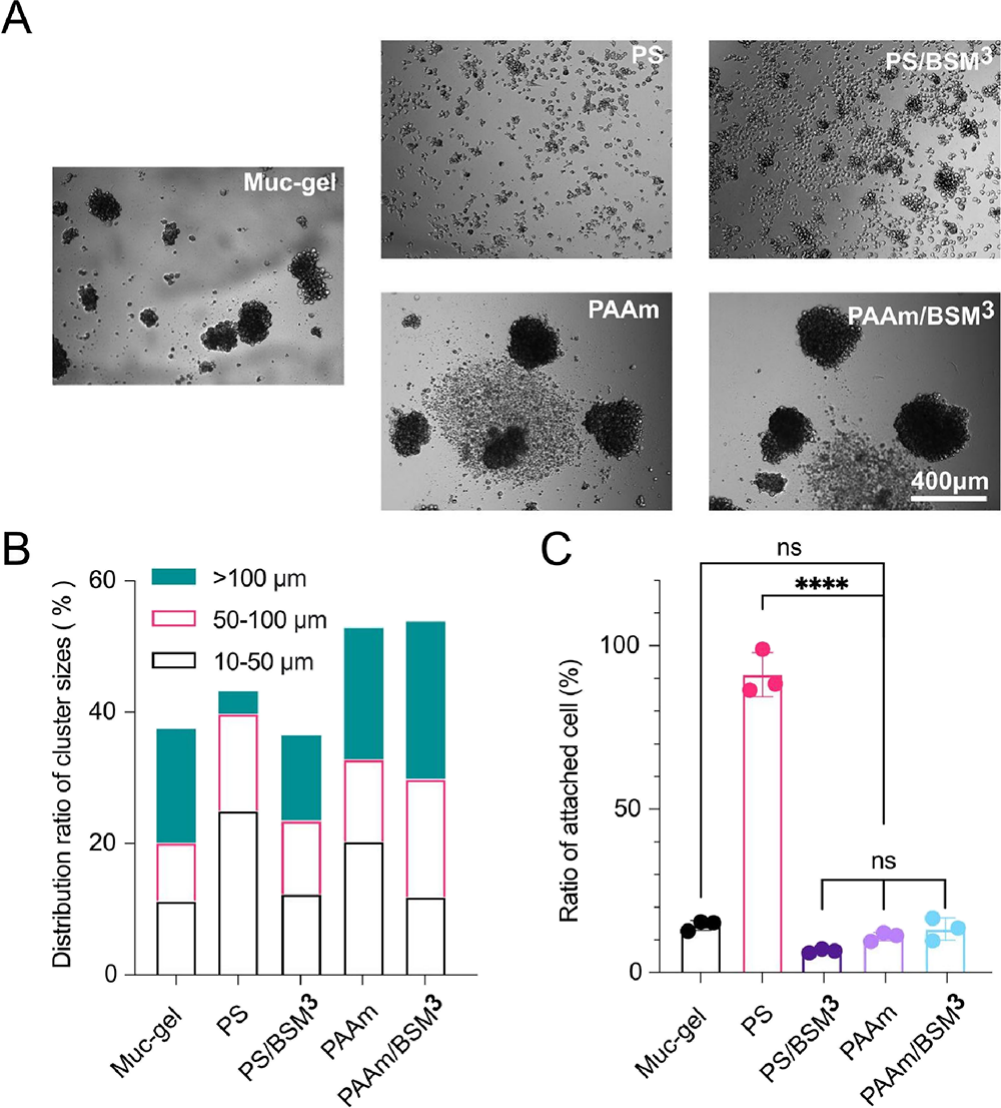
(A) Phase contrast images of macrophages after culturing on the surfaces for 1 day. (B) The distribution of normalized ratios for cell clusters with the diameter in the range of 10–50, 50 to 100, and larger than 100 μm was analyzed by a CellProfiler (4.2.0) from two independent cell experiments in triplicate. (C) Ratio of attached cells after washing with PBS as measured by DNA quantification. Statistical significance was calculated by a one-way ANOVA test by Prism (9.0). *, **, ***, and **** indicate *p* values of <0.05, 0.01, 0.0005, and 0.0001, respectively.

The attachment of macrophages on surfaces and their morphology are known to be regulated by the physical and binding properties of the material, and this in turn modulates the immune responses of macrophages.^39,40^ Here, we investigated the attachment of macrophage cells on the Muc-gel compared to Muc-film-covered PS and PAAm. Macrophages adhered to PS, and some cells became elongated on PS (Figure 3A). For the Muc-film-coated PS, the macrophages did not adhere or spread on the surface; instead, they tended to form cell clusters (Figure 3A,B). On PAAm and Muc-film-coated PAAm, most of the macrophages aggregated into large clusters, similar to those found on Muc-gel (Figure 3A,B). After washing once with PBS, more than 90% of cells were removed from the surface of Muc-gel, PS/BSM^3^, PAAm, and PAAm/BSM^3^, and on the contrary, there were approximately 90% of cells still attached on PS (Figure 3C and Figure S2A). The macrophages did not proliferate on all the materials over one day (Figure S2B), which are in agreement with previous results that showed that PMA-differentiated THP-1 cells did not proliferate.^41^ The cell metabolic activity remained constant during day 1 and increased 1.5 times on day 6, suggesting that all the material surfaces are non-cytotoxic (Figure S2C).

### 2.3 Mucin Coating Modulates Macrophage Immune Responses to PS and PAAm

**Figure 4:**
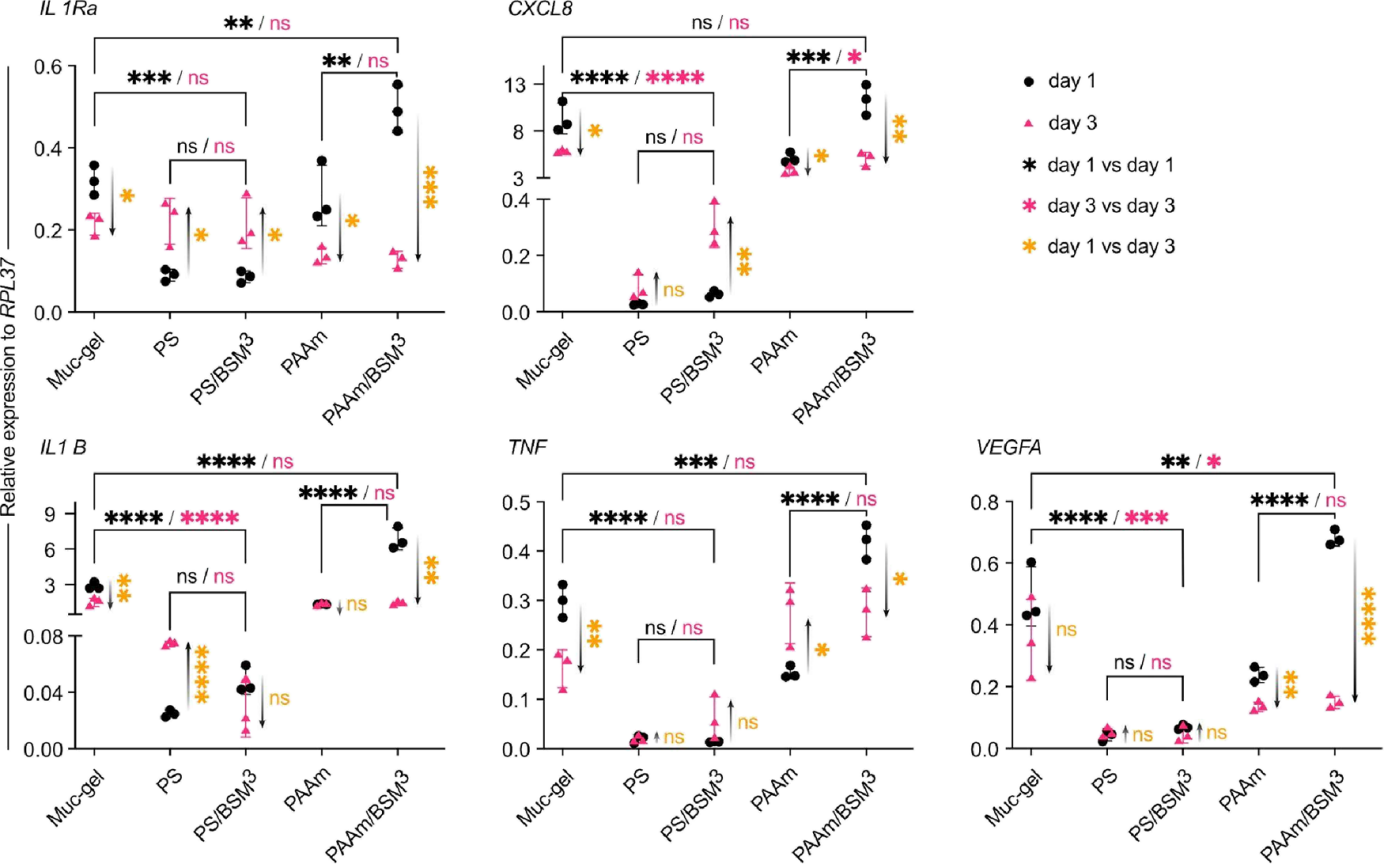
Inflammatory cytokine expression of macrophages after being cultured on the materials for one day and three days. The data dots represent the mean value of three independent cell experiments performed in duplicate. Statistical differences were calculated by a one-way ANOVA test for day 1 vs day 1 and day 3 vs day 3 and a t test for day 1 vs day 3 using Prism (9.0). *, **, ***, and **** indicate *p* values of <0.05, 0.01, 0.0005, and 0.0001, respectively, for similarities.

We then investigated whether the Muc-film coating could confer immune-modulating properties of PS and PAAm materials similar to Muc-gels. We benchmarked against the profile of strong activation of macrophage cytokine secretion after one and three days that we previously have observed for Muc-gels^10,14,26^ (Figure 4). On day 1, there was no difference between PS and PS/BSM^3^ for both the anti-inflammatory cytokine (IL1Ra) and pro-inflammatory cytokines (CXCL8, IL1B, TNF-alpha, and VEGFA) compared to Muc-gel (Figure 4) in that the values are much lower for PS and PS/BSM^3^. For PAAm at day 1, all the inflammatory cytokines of PAAm/BSM^3^ were upregulated to a similar level to that of Muc-gel (Figure 4).

After three days, there was a decrease of expression for all the cytokines for macrophages on Muc-gel, which is consistent with our previous observations.^10,14^ A similar reduction in cytokine expression was measured for macrophages on PAAm/BSM^3^ (Figure 4), whereas macrophages on unfunctionalized PAAm decreased their expression of IL1Ra, CXCL8, and VEGFA, increased the expression of TNF-alpha at day 3, and maintained high expression levels of IL1B. On day 3, macrophages cultured on PS and PS/BSM^3^ had the opposite trend compared to those cultured on Muc-gel with most of the cytokine expression (IL1Ra, CXCL8, and IL1B) increased (Figure 4).

To confirm the cytokine expression, we measured the secreted CXCL8 and TNF-alpha secreted in the cell medium (Figure S3). Similar to gene expression, macrophages on Muc-gel secreted much more CXCL8 and TNF-alpha compared to unmodified PS and PAAm over three days, and this effect only accentuated over six days. The Muc-film coating resulted in an increased secretion of CXCL8 and TNF-alpha for macrophages on PS/BSM^3^ and PAAm/BSM^3^ with TNF-alpha of PAAm/BSM^3^ increasing to the same level as that on Muc-gel on day 3 (*p* = 0.5907). Altogether, this indicates that the Muc-film coating on PAAm can confer an immune-modulating profile close to those of Muc-gels with an increased level of inflammatory cytokines on day 1 followed by dampening on day 3. In contrast, the Muc-film coating on PS was not able to confer similar bioactivity to that of Muc-gel to activate macrophages.

**Figure 5:**
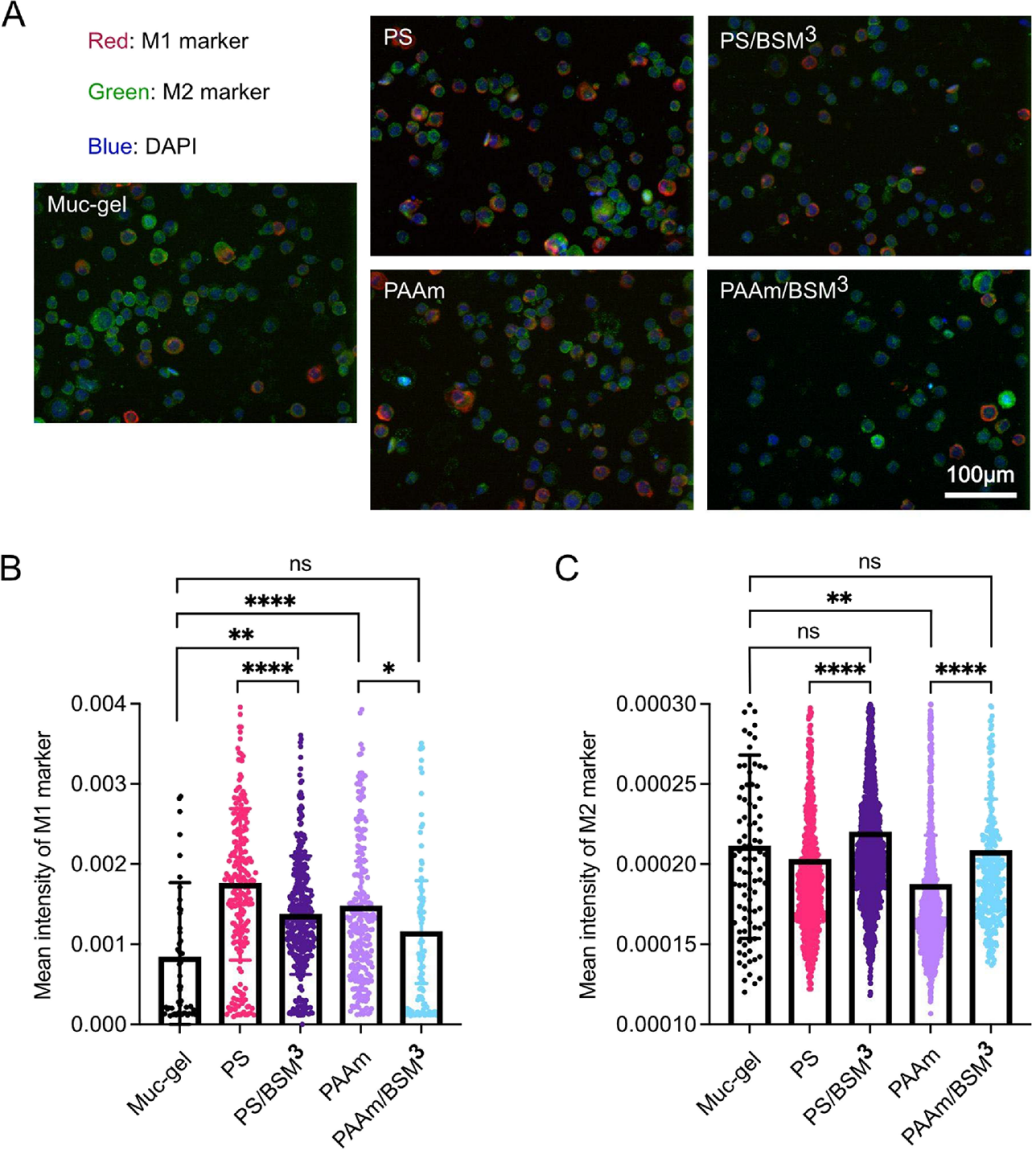
Effects of materials on macrophage phenotypes. (A) Immunostaining of calprotectin (M1 marker, in red) and the mannose receptor (M2 marker, in green) on the cell membrane and nuclei of cells labeled with DAPI (in blue). (B, C) The mean fluorescent intensities of M1 (B) and M2 (C) on cell membranes (mean fluorescent intensity = integrated fluorescent signal/cell area) were calculated using CellProfiler (v 4.2.0) based on two independent cell experiments performed in technical triplicate. Statistical differences were calculated by a one-way ANOVA test using Prism (9.0). *, **, ***, and **** indicate *p* values of <0.05, 0.01, 0.0005, and 0.0001, respectively, for similarities.

Although the density of mucin on PAAm was approximately two times less than that on PS, it showed stronger modulation of macrophage responses, suggesting that the coverage of mucins was at least sufficient to trigger a response in all conditions. To study the effect of the materials on macrophage phenotypes at a longer time, we measured the expression of pro-inflammatory phenotype (M1) and anti-inflammatory phenotype (M2) markers after six days of culture of M0 macrophages. The calprotectin (M1 marker) and the mannose receptor (M2 marker) expressed on membranes were measured by immune labeling (Figure 5). For the M1 marker, expression on uncoated PS and PAAm was about two times higher than on Muc-gel (Figure 5B) with the mean intensity of 8 × 10^–4^ for Muc-gel, 17 × 10^–4^ for PS, and 15 × 10^–4^ for PAAm. Although we obtained a good signal from the calprotectin immunolabeling, it was shown that this M1 marker mainly exists intracellularly.^42^ To confirm the effects on M1, we studied the intracellular M1 (calprotectin S100A8/A9). Similar to calprotectin gene expression, the intracellular calprotectin (Figure S4) levels kept increasing over six days and the addition of Muc-film decreased the expression of the M1 marker for both PS and PAAm. Overall, the addition of the Muc-film coating downregulated the M1 marker on both PS and PAAm. Compared to Muc-gel, the levels of the macrophage M1 marker on PS/BSM^3^ were however higher, while Muc-gel and PAAm/BSM^3^ have similar amounts of both the membrane and intracellular M1 marker.

Compared to M1, the M2 marker had the opposite trend with higher levels on Muc-gel than for macrophages cultured on the uncoated substrate, PS and PAAm (Figure 5C). After Muc-film coating, the M2 marker for both coated PS and PAAm was upregulated to the same level as in Muc-gel (Figure 5C). These results showed that the Muc-film coating on PS can also polarize macrophages but perhaps not as effectively and certainly differently compared to the Muc-gel and Muc-film coating on PAAm.

Overall, we found that the Muc-film-coated PAAm hydrogel mimics Muc-gel’s ability to modulate inflammatory cytokine expression of macrophages and the M1 and M2 phenotype polarization. The substrates were washed in the same conditions before seeding cells, and dialysis of the mucin solutions did not change the amount of endotoxin and DNA associated with BSM.^14^ Then, we assumed that the amount of endotoxin and DNA per BSM molecule stayed the same for all the mucin coatings, and the differences in the effects on macrophages were not due to differences in the type or concentrations of impurities. Although, PS could be coated with more mucin compared to PAAm (Figure 2C,D) with a similar number of multilayers of mucins in a structure likely similar to Muc-gels. It was shown that the Muc-film coating on PS mimicked the antifouling properties of Muc-gels. However, the Muc-film coating on PS cannot reproduce the effects of Muc-gel on macrophages. We hypothesize that this somewhat surprising result can be attributed to the different mechanical properties between Muc-gel and mucin coatings on PS and PAAm. The stiffness of hydrogels has previously been shown to affect the macrophage morphology^43^ and gene expression.^44−46^ Such effects of mechanical properties can act in synergy with chemical sensing through binding of cell surface receptors. Similar synergies have also been shown for integrin binding and bone morphogenetic protein receptors.^47−49^

In addition to the underlying substrate stiffness, it could be said that the interactions between PS and mucin might induce limited accessibility of mucin glycan ligands to the surface receptors on the macrophages. Lectin binding to mucin-coated PS showed differences between the covalently grafted mucins and the non-covalently grafted mucin, regarding the binding affinity of peanut agglutinin (PNA) and wheat germ agglutinin (WGA) (Figure S5). Since the lectin molecules are small in size, they can diffuse into Muc-gel and PAAm hydrogels, such mucin glycan accessibility could however not be studied in this work. To overcome this limitation, cell-mimicking beads with immobilized lectins could be developed in a future study to evaluate the accessibility of glycans to specific cell receptors.

## 3 Conclusions

With this study, we show that the immune-modulating functions of mucins can be strongly affected when functionalized (e.g., adsorbed or grafted) onto substrates. We highlight that the chemical and mechanical properties of the substrate determine the bioactivity of the mucins toward macrophages and only Muc-film coated on soft PAAm could mimic the properties of Muc-gel. This implies that it is theoretically possible to confer mucin immune-modulating properties to a broad range of biomaterials by simply adding a thin mucin coating, albeit if the mechanical properties are well considered. On hard surfaces, other grafting strategies are needed, and providing more flexibility to the mucin molecules could compensate for the limitation seen in this study. Beyond the direct implications for the development of immune-modulating biomaterials, this study also poses the question of the importance of mechanical properties in the immune-modulating function of mucin in other physiological contexts, such as on the surfaces of tumors and parasites.^50,51^

## 4 Materials and Methods

### 4.1 Materials

Bovine submaxillary mucin (BSM, M3895-1G, lot no. SLCC4979) was purchased from Sigma-Aldrich, and it was purified further before using. The BSM was dissolved in MQ water at 4 °C overnight, and then the solution was ultracentrifuged at 150,000*g* for 1 h at 4 °C. The supernatant was separated from the pellet carefully and lyophilized for applications in this project. Tetrazine amine (Tz, CP-6021) was purchased from Conju-Probe, LLC. 5-Norbornene-2-methylamine (Nb, mixture of isomers, N0907) was ordered from TCI EUROPE N.V. *N*-[(3-Trimethoxysilyl) propyl] ethylenediamine triacetic acid trisodium salt (TMS-EDTA) was ordered from abcr, Germany. Human monocytes (THP-1) were purchased from ATCC. The medium for cell culture and reagents for real-time PCR were purchased from Thermo Fisher Scientific. The RNA purification kit (RNeasy Mini, 74104) was ordered from Qiagen, and other chemicals were obtained from Sigma-Aldrich.

### 4.2 Modifying BSM with Tetrazine and Norbornene

The chemistry process of conjugating Tz and Nb onto BSM was adapted from previous studies.^10,26^ Briefly, BSM of 10 mg/mL was dissolved in 100 mM MES buffer with 300 mM NaCl of pH 6.5 at 4 °C overnight. *N*-Hydroxysuccinimide (NHS) and 1-ethyl-3-(3-dimethylaminopropyl) carbodiimide (EDC) were added into the BSM solution to a final concentration of 50 mM and incubated for 30 min at room temperature to activate carboxyl groups. Then, Tz (1 mmol/1 gram BSM, dissolved in DMSO) and Nb (2 mmol/1 gram BSM) were added, and the reactions were incubated at 4 °C overnight. The products were cleaned up by dialysis (MWCO 100 kDa, Spectra-Por Float-A-Lyzer G2) first against 4 L of 300 mM NaCl for two days and then against MQ water for one day at 4 °C. Products were lyophilized and stored at −80 °C, and the products were named as BSM-Tz and BSM-Nb.

### 4.3 Mucin Covalent Coating and Multilayer Assembly on Polystyrene

To introduce carboxylic acid groups on polysterene for coupling mucin, we adapted a reported protocol^21^ to modify the surface with carboxylated silane. Polystyrene microplates (Sarstedt, 82.1581) were activated in a plasma etching machine (Plasma chamber Pico, Diener electronic) with air at 0.4 mbar under 50 W of power for 60 s. Each well was then covered immediately with 100 μL of TMS-EDTA (0.1%, *w*/*v*) in 10 mM acetate buffer (pH 4.5) and then incubated for 4 h at 60 °C. After the incubation, each well was washed three times with 80% ethanol at 60 °C for 20 min. The carboxyl groups on each well were activated with 100 μL of EDC/NHS at 5 mM in MES buffer (150 mM, pH 5.0) for 30 min at room temperature. Then, 50 μL of BSM-Tz (1 mg/mL) in PBS of pH 7.4 was added and kept under 4 °C overnight. To assess the stability, the coatings were washed with 200 μL of PBS three times and kept in PBS overnight at 4 °C. For multilayer assembly via a click reaction between tetrazine and norbornene groups, 50 μL of BSM-Nb or BSM-Tz solution (1 mg/mL) in PBS of pH 7.4 was added and incubated at room temperature for 2 h. The coating was washed three times with 200 μL of PBS and kept in PBS overnight at 4 °C after each layer assembly. The resulting materials are referred to as PS/BSM^*n*^ with “*n*” indicating the number of layers assembled via a click reaction. As a comparison, a non-covalent coating of mucin was generated by adding 50 μL of BSM-Tz (1 mg/mL) in PBS buffer of pH 7.4 onto the same PS plate without EDC/NHS activation, and it was also kept under 4 °C overnight. The same washing process as that of the covalently grafted coatings was performed. The resulting materials are referred to as PS#BSM^*n*^, and “*n*” indicates the number of adsorption sequences.

### 4.4 Polyacrylamide Gel Preparation and Mucin Coating on the Gels

The chemical reaction was adapted from the protocol developed in Yu-Li Wang’s Laboratory.^52^ To couple mucin, acrylic acid (AA, 100%) was introduced to co-polymerize with acrylamide.^53^ In brief, a 40% (*w*/*v*) acrylamide/bis-acrylamide (29:1, Bio-Rad, 3.3% cross-linker) solution was degassed and then mixed with AA, degassed MQ, and 1 M HEPES buffer of pH 7.0 to the final concentrations of AA at 0.4% (*v*/*v*), acrylamide at 5% (0.17% bis-acrylamide), and 0.1 M HEPES buffer. Then, 1% (*v*/*v*) ammonium persulfate (APS, 10 mg/mL in degassed MQ) and 0.1% (*v*/*v*) *N*,*N*,*N*′,*N*′-tetramethylethylenediamine (TEMED) were then added into the solution to initiate the polymerization. The TMS-EDTA-modified polystyrene plate was used as a substrate for PAAm gels. To minimize the inhibition effect of oxygen on the polymerization, the plates were placed in a dessicator with a constant nitrogen flow 2 h before the mucin grafting. A volume of 100 μL of the reaction solution (containing AA, acrylamide, APS and TEMED) was added into each well, and the plate was immediately put back into the dessicator with flowing nitrogen, and it was left at room temperature for 2 h to complete the polymerization reaction.

The Young’s modulus of 5% PAAm hydrogel cross-linked with 0.12% bis-acrylamide from Yu-Li Wang’s Laboratory is 33 kPa, which equal to approximately 11 kPa of storage modulus based on the equation *G* = *E*/2(1 + *v*). *v* is the Poisson ratio of PAAm, and it is around 0.5.^54^ The result is in agreement with the reported storage modulus of PAAm of approximately 10 kPa.^55^ The storage modulus of the Muc-gels (25 mg/mL) was reported to be approximately 10 kPa,^10,13,14^ and only one batch of the Muc-gel was reported to be 205 kPa.^26^ Comparing with the 3 GPa of elastic modulus for polystyrene,^56^ we then considered the PAAm as a model “soft” material within the range of Muc-gels and PS as a model “hard” material in the range of other solid implantable biomaterials made of metals, ceramics, or polymers.

Before mucin coating, PAAm was washed with 200 μL of MES buffer (150 mM, pH 5.0) 10 times, and then the carboxyl groups on the surface of the gels were activated with EDC/NHS under the same conditions as coating on PS. The same mucin covalent grafting and multilayer assembly with PS was performed for PAAm gels. The resultant materials were labeled as PAAm/BSM^*n*^, and the superscript “*n*” of BSM represents the number of mucin layers assembled via a click reaction.

### 4.5 Quantification of the Mucin Amount in Coatings by Fluorescence Intensity

BSM-Tz and BSM-Nb were first labeled with Fluorescein isothiocyanate (FITC). Purified BSM-Tz or BSM-Nb was first dissolved at 5 mg/mL in sodium carbonate buffer (0.1 M, pH 9.0) under 4 °C overnight. FITC in DMSO at 10 mg/mL was then added into the solution under a vortex with a *v*/*v* ratio of 1:40. The mixture was incubated for 1.5 h at room temperature and then dialyzed (MWCO 100 kDa, Spectra-Por Float-A-Lyzer G2) against 300 mM NaCl for 2 days and then against MQ water for 1 day. The samples were lyophilized and stored at −80 °C before use. For studying the coating process, the FITC-labeled BSM-Tz or BSM-Nb were used for the coating under the same conditions as the non-labeled mucin, and the whole process was protected from light by an aluminum film. Standard curves with different concentrations of FITC-labeled BSM-Tz and BSM-Nb in the same plate were employed to quantify the mucin amount. The fluorescence of the coatings and standard FITC-labeled BSM-Tz or BSM-Nb solution were read by a plate reader (Clario Star, BMG Labtech). A measurement of 0.32 cm^2^ was used as the area for each well to calculate the amount of mucin in the coatings. To study the stability of mucin coatings, the coatings were washed daily in PBS with 0.02% NaN_3_ at 37 °C for one week. The fluorescence intensity was measured at each time point, and the percentage of mucin left after incubation was normalized by the fluorescence intensity of the first day.

### 4.6 Fluorescence Imaging of FITC-Labeled Mucin on PS and the PAAm Hydrogel

FITC-labeled BSM-Tz was covalently grafted on PS as described above (PS/BSM^1^), and the coated surface was washed with PBS for two days at 4 °C before fluorescent imaging. Scratches on the coating were made by a pipette tip to show the difference between the PS substrate and mucin coating. Fluorescent images of PS/BSM^1^ were taken with a fluorescence microscope (Nikon Eclipse Ti, equipped with CoolLED pE-300) with a 20× objective. For PAAm, we first labeled this with Alexa Fluor 647 hydroxylamine (ThermoFisher, A30632) via 5 mM EDC/NHS (in PBS of pH 7.4) coupling, and the molar ratio between Alexa Fluor 647 hydroxylamine and acrylic acid in PAAm is 1:100 to get enough carboxylic acid groups to remain for grafting mucin. The labeled PAAm was washed with PBS for two days at room temperature, and then FITC-labeled BSM-Tz was covalently grafted on the labeled PAAm as described above (PAAm/BSM^1^). The cross section of PAAm/BSM^1^ was imaged with a confocal microscope (Zeiss LSM 900-Airy2) with a 20× objective.

### 4.7 Culture of THP-1 and Differentiation into Macrophages

An RPMI-1640 medium containing 10% FBS and penicillin/streptomycin (100 U/mL) was labeled as the complete medium, and THP-1 was cultured in the complete medium in a humidified incubator with 5% CO_2_ at 37 °C. Then, the cells were differentiated into macrophages by culturing THP-1 in the complete medium with phorbol 12-myristate 13-acetate (PMA, 150 nM) for three days, and then the medium was changed to the complete cell medium for at least 12 h before seeding onto different surfaces. After differentiation, the THP-1 suspension adhered on the tissue culture petri dish. As in our former work, we confirmed the presence of differentiated macrophages by checking macrophage markers.^14^ The macrophage was detached by incubating in Accutase for 10 min and washed with the complete medium once. A volume of 100 μL of macrophages (10^5^ cells/mL) was seeded onto the surfaces of PS, PAAm, and 25 mg/mL Muc-gel. BSM-Tz or BSM-Nb without FITC labeling was used for coatings interacting with cells. All the surfaces were sterilized by UV for 1 h at room temperature and then washed with the complete cell medium at 4 °C for two days before seeding cells.

### 4.8 Cell Clustering and Surface Attachment

Phase contrast images of macrophages cultured on the surfaces for one day were taken with a fluorescence microscope (Nikon Eclipse Ti, equipped with CoolLED pE-300). Images from the same positions of each well were taken, and then the cell clusters were analyzed by a CellProfiler (4.2.0) based on two independent cell experiments in triplicate for each sample. To study the cell attachment after culturing on the surfaces for one day, the cell medium was taken out, and then the well was washed with 100 μL of PBS without mixing and taken out immediately. The remaining cells on the surfaces were lysed with a lysis buffer, and then the DNA amount was measured by a Qubit 1× dsDNA HS Assay Kit (ThermoFisher, Q33230) under the provided instructions of Qubit 4. The DNA ratio between the washed surface and unwashed surface was calculated, and it is applied as the ratio of attached cells.

### 4.9 Real-Time PCR for Gene Expression

After seeding THP1-M0 on materials for one day and three days, cells were detached from the surfaces by pipetting up and down 10 times, and then cells were transferred into Eppendorf tubes and collected by centrifuge at 400*g* for 5 min at room temperature. The RNA was extracted following the provided protocol from an RNeasy mini kit (Qiagen), and then cDNA was synthesized using Superscript III polymerase (Invitrogen). The gene expression of inflammatory cytokines was measured by real-time PCR (CFX96 Touch, Bio-Rad) and TaqMan probes for specific cytokine. *RPL37* was employed as the reference gene for the THP-1-derived macrophage.

### 4.10 Immunofluorescence Staining

Cells were washed off the surfaces and transferred into 1.5 mL Eppendorf tubes. The cells were collected by centrifuging at 400*g* for 5 min, and then cells were re-dispersed in 100 μL of PBS after washing with 1% goat serum in PBS. Immunostaining of cells was performed in a 96-well plate with a high-performance cover glass bottom (P96-1.5H-N, Cellvis). To get the cells to adhere on the glass bottom of the plate, the glass was coated with polylysine by adding 50 μL of polylysine (0.01%, 70 kDa to 150 kDa, Sigma) into each well. The coating was incubated at room temperature for 1 h and washed with MQ once. A volume of 100 μL of cell solution was added into each polylysine coated well, and the plate was centrifuged at 400*g* for 10 min at 4 °C, and then the supernatant was taken out gently. To fix the cells, 100 μL of 4% paraformaldehyde in PBS was added into each well and incubated at room temperature for 10 min, and the cells were washed with PBS carefully after fixation. Two steps of blocking were performed to avoid nonspecific binding of antibodies, including bovine serum albumin (BSA, 3% in PBS with 1% glycine) at room temperature for 1 h and then normal goat serum in PBS (5%, ab7481 from abcam) at room temperature for another 1 h. The cells were then labeled with mouse anti-human calprotectin immunoglobulin G1 (2 μg/mL in 1% goat serum, S100A9, MA181381 from Invitrogen) and a rabbit anti-mannose receptor antibody (MR, 1 μg/mL in 1% goat serum, ab64693 from abcam) for 1 h at room temperature. After rinsing with PBS twice, cells were stained with secondary antibodies at room temperature for another 1 h with mixture of Rhodamine-X goat anti-mouse IgG (H + L) (8 μg/mL, R6393 from Invitrogen) and Alexa Fluor 488 goat anti-rabbit IgG (H + L)(8 μg/mL, A11008 from Invitrogen) in 1% goat serum. The cell nuclei was labeled with DAPI (0.5 μg/mL) for 5 min and then washed by PBS. Cells were imaged with 100 μL of PBS in each well. Cells were imaged automatically by a microscope with a 20× objective (ImageExpress Pico from Molecular device). The density of MR and calprotectin across the whole membrane of each cell (mean fluorescent intensity = integrated fluorescent signal/cell area) were measured by a CellProfiler (4.2.0) for cell images from two independent experiments in triplicate.

## Supporting Information Available

Quantification of norbornene and tetrazine grafting by NMR, indentation by atom force microscopy with a colloidal probe, cell viability and proliferation on the coatings, ELISA of cytokines and calprotectin, and lectin binding to mucin coatings on polystyrene surfaces (PDF)
