# Supplementary material for: Engineering Surfaces with Immune Modulating Properties of Mucin Hydrogels
Source: ACS Appl Mater Interfaces. 2022 Aug 24;14(35):39727–35. doi: 10.1021/acsami.1c19250 (PMC9460428; doi:10.1021/acsami.1c19250)
Supplement: Supplementary file 1 — am1c19250_si_001.pdf [file am1c19250_si_001.pdf]

Supporting Information

Engineering surfaces with the immune modulating properties of mucin hydrogels

Kun Jiang[1,2,3]*, Xueyu Wen*[1]*, Torbjörn Pettersson*[4]*, Thomas Crouzier**[1,2,3]

[1]Division of Glycoscience, Department of Chemistry, School of Engineering Sciences in Chemistry, Biotechnology and Health, KTH, Royal Institute of Technology, AlbaNova University Center, 106 91 Stockholm, Sweden

[2]AIMES - Center for the Advancement of Integrated Medical and Engineering Sciences at Karolinska Institutet and KTH Royal Institute of Technology, Stockholm, Sweden

[3]Department of Neuroscience, Karolinska Institutet, SE-171 77, Stockholm, Sweden

[4]Department of Fibre and Polymer Technology, KTH Royal Institute of Technology, SE-100 44 Stockholm, Sweden

Keywords: biomaterials, mucin coating, immune-modulating, macrophage polarization

Quantification of norbornene and tetrazine grafting by NMR

The quantification was performed as described in the former paper[1]. Mucin samples (S, mg) were dissolved in 0.6 mL deuterium oxide (around 30 mg/mL) containing 30 mM maleic acid. ^{1}H-NMR spectra were obtained with a Bruker Ultrashield plus 500 MHz spectrometer (Bruker Corporation, USA) and the data was processed with the MestReNova Software (version 12.0.4-22023). 6.285 ppm was selected as maleic acid peak, which is the shift of the two protons attached to the alkene and the integration area is labeled as M. Peaks within 7.15 to 7.7 ppm belong to the two protons on benzene far away from the tetrazine ring and 5.8 to 6.2 ppm

belong to the 2 protons attaching to sp2 alkene in norbornene. The integration areas within 7.15 to 7.7 ppm and 5.8 to 6.2 ppm were labeled as T and N. The quantity of tetrazine (umol/mg) on BSM (Qt) and norbornene (μmol/mg) on BSM (Qn) are calculated by equations:$Qt = \frac{T \times 18}{M \times S}$ and $Qn = \frac{N \times 18}{M \times S}$; the 18 indicates the 18 μmol maleic acid in the solution.

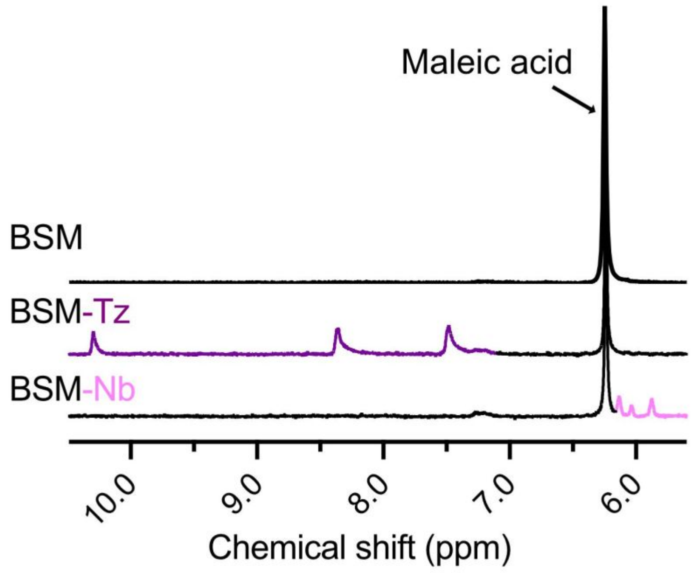

Figure S1. The NMR spectrums of BSM and functionalized BSM. The peak of tetrazine and norbornene on mucin confirmed the successful grafting, and the indicated peak of maleic acid is used for quantifying the amount of tetrazine and norbornene on mucin.

Table S1. The quantity of tetrazine and norbornene on BSM by qNMR and the number of Tz or Nb per BSM molecule calculated by taking the molecular weight of BSM-Tz or -Nb as 1 MDa[1].

Sample	BSM-Tz	BSM-Nb
Tz or Nb on BSM (µmol / mg)	0.15 ± 0.04	0.10 ± 0.03
Tz or Nb on BSM (Number per BSM molecule)	150± 4	100± 3

Indentation by atom force microscopy with colloidal probe

The elastic modulus of the substrates was measured by an atom force microscope (AFM, MultiMode 8, Bruker, CA, USA) equipped with a Picoforce extension. The substrate samples were glued on a magnetic metal to stabilize them from lateral motion during the measurement. The indentation was performed in a liquid cell in PBS of pH 7.4 for all the substrates. A tipless rectangular cantilever (CLFC-NOCAL, Brucker) with a normal spring constant of about 0.24 N/m was employed and a borosilicate glass sphere (radius = 3 µm) was glued on the cantilever as the indentation probe[2,3]. The exact spring constant of the cantilever was determined using the AFM Tune IT (ForceIT, Sweden) employing the calibration method based on thermal noise with hydrodynamic damping[4]. To calculate the indentation force, the deflection sensitivity was obtained by force measurements on clean silicon wafers before and after measuring each substrate. Hertz model in linearized form was fitted to the force vs separation curves[5,6] (in the force range of 10 nN to 30 nN) and a poisson ratio of 0.47 was employed to calculate elastic modulus for all the substrates[7,8]. The average modulus and standard deviation were calculated based on at least 30 measurements on each sample.

Table S2. The elastic modulus of substrates measured by Atom Force Microscopy indentation.

Sample	Muc-gel	PS	PS/BSM[3]	PAAm	PAAm/BSM[3]
Elastic modulus (kPa)	31.5 ± 1.8	354000 ± 119000	264000 ± 134000	12.7 ± 2.5	7.1 ± 0.8

Cell viability and proliferation on the coatings

The viability of cells was analyzed by alamarBlue™ Cell Viability Reagent (Invitrogen, DAL1025). 10 µL alamarBlue was added into each well (100 µL cell medium) after culturing macrophages on the surfaces for 2 h, 1 day and 6 days, and the cell viability of 2 h was used as the reference value to calculate the relative cell viability on day 1 and day 6. After adding alamarBlue, cells were put back into the humidified incubator with 5% CO_2 at 37 °C and incubated for 4 h before reading fluorescence (excitation at 544 nm and emission at 590 nm) with a plate reader (Clario Star, BMG Labtech). The DNA amount was measured by the Qubit™ 1X dsDNA HS Assay Kit (ThermoFisher, Q33230) following the manufacturer's instructions with Qubit 4. The ratio of total DNA content of cell cultures between 2 h after seeded and after 1 day.

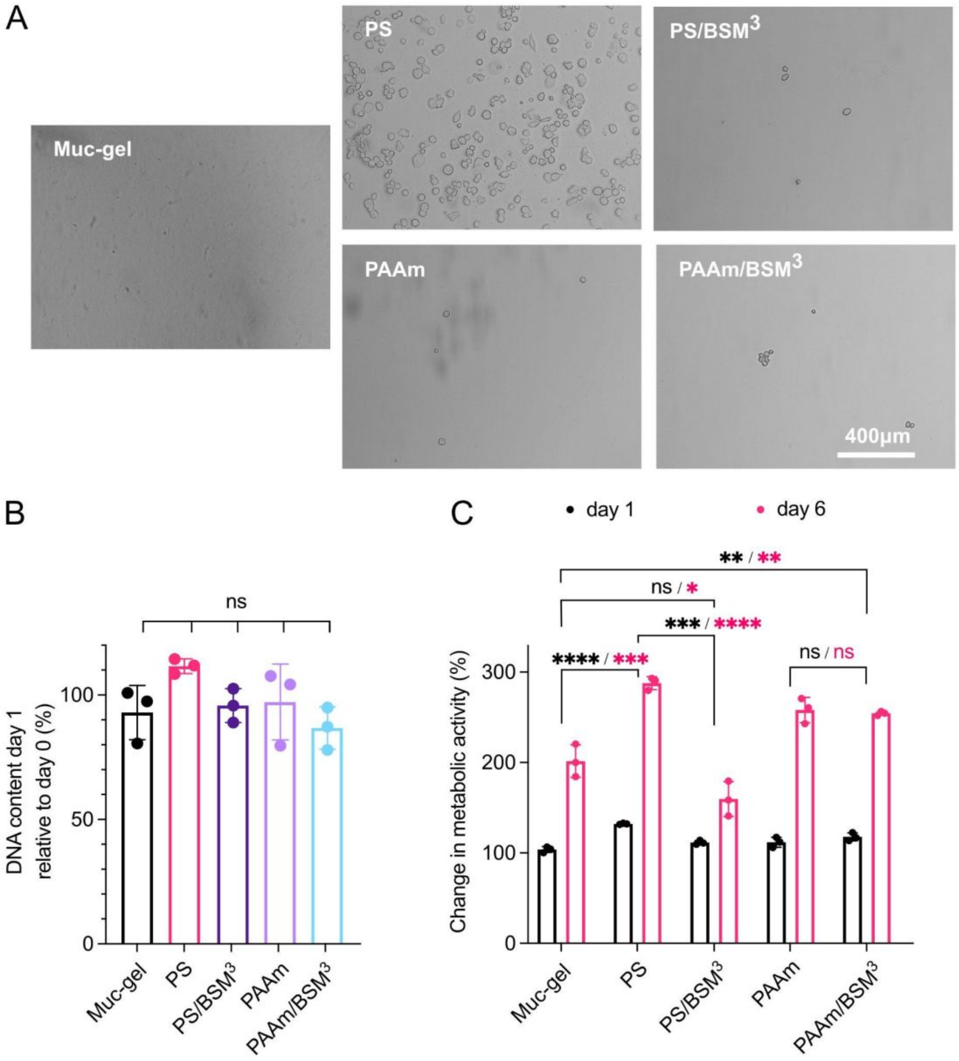

Figure S2. Phase contrast images of cells on mucin coatings after washing once with PBS (A), DNA content of cell cultures on day 1 relative to that on day 0 (B), and the cell metabolic activity of cells culturing on the mucin coating for 1 day and 6 days as measured by AlamarBlue (C). Statistical differences were calculated by one-way ANOVA test by Prism 9.0. *, **, ***, and **** indicate p values of <0.05, 0.01, 0.0005, and 0.0001, respectively.

ELISA of cytokines and calprotectin

Secreted cytokines in cell medium were quantified by an ELISA kit according to the protocol provided by the supplier (*TNF-alpha* (Invitrogen, 88-7346-22), *CXCL8* (abcam, ab214030). Intracellular calprotectin (S100A8/A9) was quantified by an ELISA kit following the

procedure from the supplier (proteintech, KE00177). Briefly, after culturing THP1-M0 on the materials for 1, 3, and 6 days, cells were detached from the surfaces by pipetting up and down 10 times. Then cells were collected into Eppendorf tubes and were centrifuged at 400g for 5min at room temperature. The supernatant was taken out carefully and stored at -80°C before performing the ELISA assaying secreted cytokines. The cell pellet was washed with PBS and then was lysed by a mammalian protein extraction reagent (Thermo Scientific, 78503). The calprotectin in the cell lysis was measured by ELISA. The protein concentration in cell lysis was assayed by the Pierce BCA protein assay kit (Thermo Scientific, 23225). Results are expressed in pg/mL after normalizing by the normalized value of cell lysis protein.

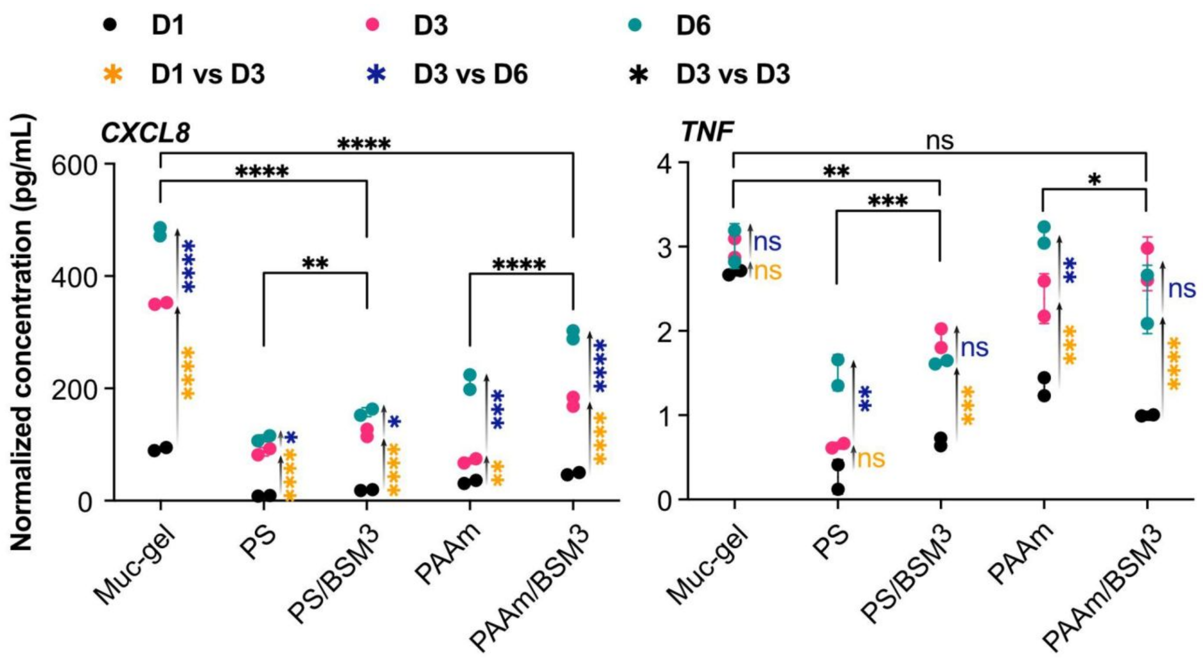

Figure S3. Secreted cytokines (*CXCL8* and *TNF-alpha*) of macrophage after culturing on materials on 1 day, 3 days, and 6 days. Statistical differences were calculated by a two-way ANOVA test using Prism 9.0. *, **, ***, and **** indicate *p* values of <0.05, 0.01, 0.0005, and 0.0001, respectively for similarities.

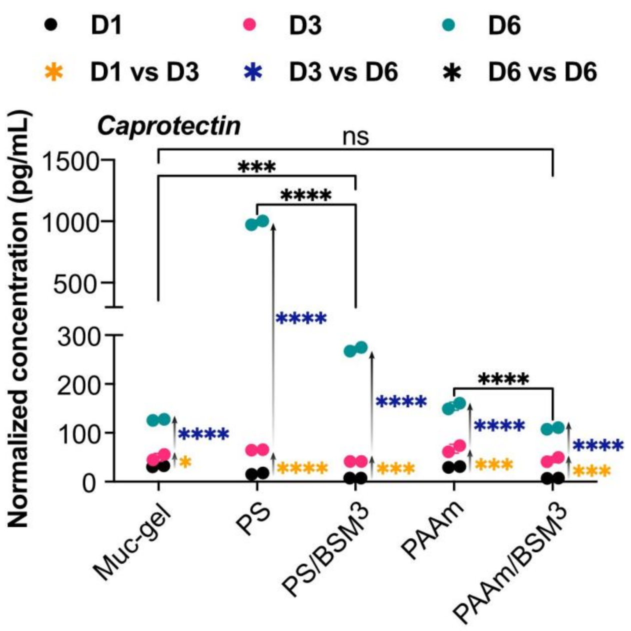

Figure S4. Intracellular M1 marker (calprotectin, S100A8/A9) of macrophage after culturing on materials on 1 day, 3 days, and 6 days. Statistical differences were calculated by a two-way ANOVA test using Prism 9.0. *, **, ***, and **** indicate *p* values of <0.05, 0.01, 0.0005, and 0.0001, respectively for similarities.

Lectin binding to mucin coatings on PS

Non-labeled mucin was coated on PS via covalent bonds in the same way as described in the experimental section. The coatings were washed with PBS at 4°C for 2 days before the lectin binding experiment. Each coating was blocked with 100 µL BSA (20 mg/mL in PBS) for 30 min at room temperature to block non-specific lectin binding. Then 50 µL fluorescence labeled lectin solution (20 µg/mL in PBS, VECTOR Laboratories, FLK-2100) was added to each well and incubated at room temperature for 3h. The surfaces were washed with PBS for 3 times and 50 µL PBS was added into each well before reading fluorescence (excitation at 483 nm and emission at 530 nm) with a plate reader (Clario Star, BMG Labtech). The following lectins were chosen to the study the accessibility of target glycans on mucin: peanut agglutinin (PNA)

which preferably binds galactose, wheat germ agglutinin (WGA) which preferably binds N-acetylglucosamine, ulex europaeus agglutinin I (UEA) which preferably binds fucose, and ricinus communis agglutinin I (RCA) which preferably binds galactose and N-acetylgalactosamine. The fluorescence intensity of bond lectin was normalized by the mucin coating amount measured by the FITC labeled mucin coating (**Figure 2C**) to indicate the glycan accessibility.

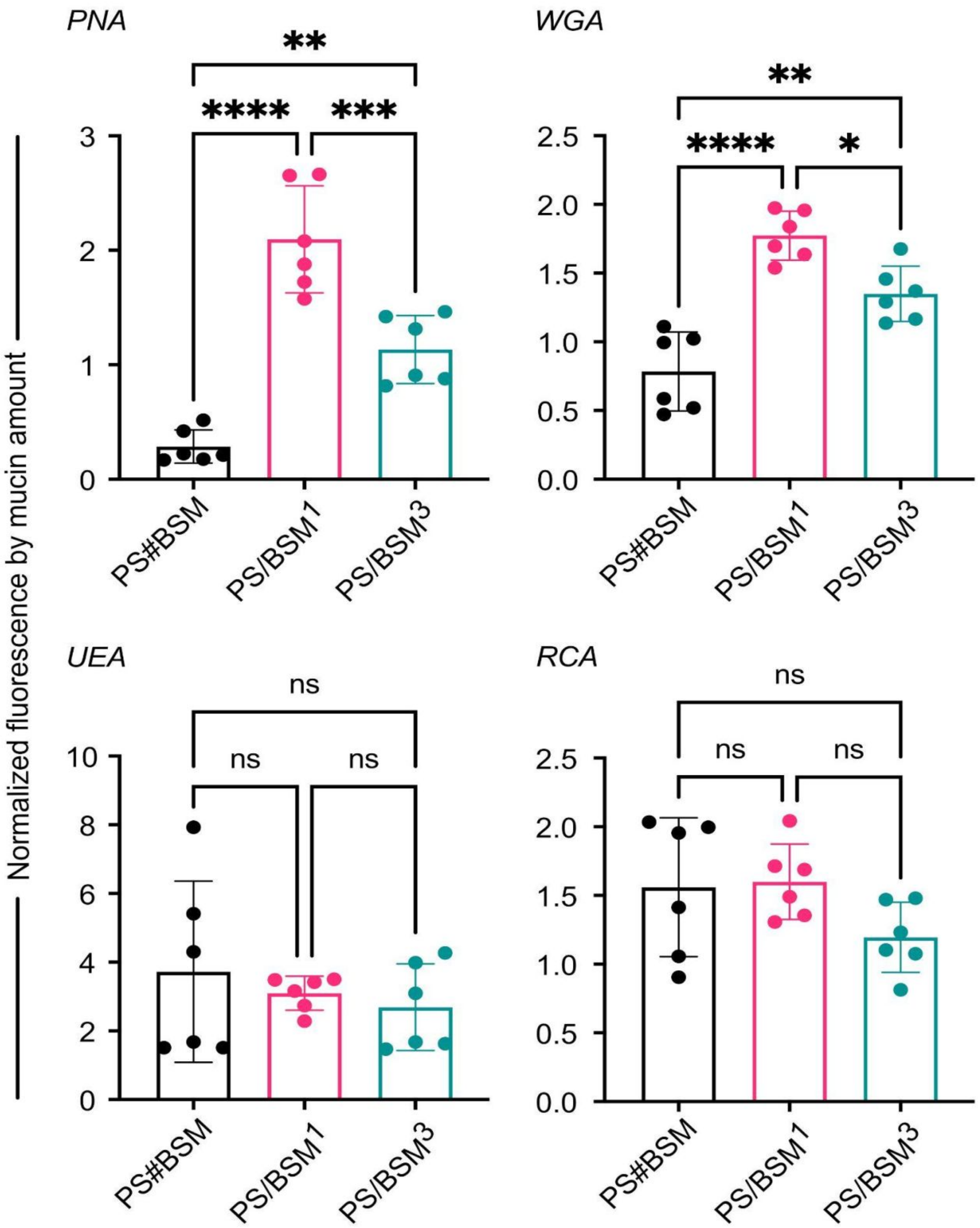

Figure S5. Fluorescence of lectin bound to mucin coatings and multilayer films, normalized to the amount of mucin in each coating. The '/' indicate the covalent bond between substrate and mucin, and the '#' indicates the physical interaction between substrate and mucin. The superscript 'n' of BSM represent the number of layers assembled via click reaction. Statistical differences were calculated by one-way ANOVA test by Prism 9.0. *, **, ***, and **** indicate p values of <0.05, 0.01, 0.0005, and 0.0001, respectively.

References

(1) Jiang, K.; Yan, H.; Rickert, C.; Marczynski, M.; Sixtensson, K.; Vilaplana, F.; Lieleg, O.; Crouzier, T. Modulating the Bioactivity of Mucin Hydrogels with Crosslinking Architecture. *Adv. Funct. Mater.* **2021**, 2008428.

(2) Pettersson, T.; Feldötö, Z.; Claesson, P. M.; Dedinaite, A. The Effect of Salt Concentration and Cation Valency on Interactions Between Mucin-CoatedHydrophobic Surfaces. In *Surface and Interfacial Forces – From Fundamentals to Applications*; Springer Berlin Heidelberg, 2008; pp 1–10.

(3) Ducker, W. A.; Senden, T. J.; Pashley, R. M. Direct Measurement of Colloidal Forces Using an Atomic Force Microscope. *Nature* **1991**, *353* (6341), 239–241.

(4) Sader, J. E.; Chon, J. W. M.; Mulvaney, P. Calibration of Rectangular Atomic Force Microscope Cantilevers. *Rev. Sci. Instrum.* **1999**, *70* (10), 3967–3969.

(5) Carl, P.; Schillers, H. Elasticity Measurement of Living Cells with an Atomic Force Microscope: Data Acquisition and Processing. *Pflugers Arch.* **2008**, *457* (2), 551–559.

(6) Hellwig, J.; Durán, V. L.; Pettersson, T. Measuring Elasticity of Wet Cellulose Fibres with AFM Using Indentation and a Linearized Hertz Model. *Anal. Methods* **2018**, *10* (31), 3820–3823.

(7) Takigawa, T.; Morino, Y.; Urayama, K.; Masuda, T. Poisson's Ratio of Polyacrylamide (PAAm) Gels. *Polym. Gels Networks* **1996**, *4* (1), 1–5.

(8) Pierscionek, B. K.; Asejczyk-Widlicka, M.; Schachar, R. A. The Effect of Changing Intraocular Pressure on the Corneal and Scleral Curvatures in the Fresh Porcine Eye. *Br. J. Ophthalmol.* **2007**, *91* (6), 801–803.
